# Supplementary material for: The genome sequence of an ichneumonid wasp, Ophion ventricosus Gravenhorst, 1829
Source: Wellcome Open Res. 2024 Aug 15;9:481. [Version 1] doi: 10.12688/wellcomeopenres.22874.1 (PMC11544200; doi:10.12688/wellcomeopenres.22874.1)
Supplement: Peer review history

# Reviewer report: Reviewer response for version 1

**DOI:** 10.21956/wellcomeopenres.25189.r109602

**Published:** 2025-01-16

**Authors:** Marko Mutanen

**Affiliations:** 1 University of Oulu, Oulu, Finland

The genome is very valuable as concerns a diverse group that has seen significant taxonomic revisions lately (e.g. Johansson et al.). The genome for *O. ventricosus* hopefully will become of much use study the cryptic diversity of these parasitic wasps in different areas of the world.

 The Introduction gives a nice short overview of the group and this particular species. I appreciate that the focus in not limited to British Isles only but a related extralimital species is mentioned too. I find it a strength that the identity of this species was confirmed by DNA barcoding.

 The genome is evidently of high quality as indicated for example by the high coverage of reads and high k-mer completeness. That the BUSCO coverage does not exceed the 95% benchmark undoubtedly is not an indication of poor quality of the genome, but rather suggests that in this species many of the 5,991 BUSCO loci truly are not present in this species.

Are sufficient details of methods and materials provided to allow replication by others?

Yes

Is the rationale for creating the dataset(s) clearly described?

Yes

Are the datasets clearly presented in a useable and accessible format?

Yes

Are the protocols appropriate and is the work technically sound?

Yes

Reviewer Expertise:

Molecular taxonomy, phylogenetics, Lepidoptera, Sawflies, DNA barcoding, biodiversity genomics, dark taxa

I confirm that I have read this submission and believe that I have an appropriate level of expertise to confirm that it is of an acceptable scientific standard.

# Reviewer report: Reviewer response for version 1

**DOI:** 10.21956/wellcomeopenres.25189.r109600

**Published:** 2024-11-13

**Authors:** Eric Ganko

**Affiliations:** 1 Syngenta Biotechnology Inc, Research Triangle Park, NC, USA

This note details a new, unphased genome sequence for the parasitoid wasp *Ophion ventricosus* using PacBio and Hi-C data. The rationale and pipelines for genome assembly have been thoroughly documented by the Darwin Tree of Life Consortium. Assembly quality is acceptable with good pseudomolecule representation (97%)and reasonable Busco scores (especially at the higher taxa levels, i.e. insecta). Overall, this report is ready for indexing.

Are sufficient details of methods and materials provided to allow replication by others?

Yes

Is the rationale for creating the dataset(s) clearly described?

Yes

Are the datasets clearly presented in a useable and accessible format?

Yes

Are the protocols appropriate and is the work technically sound?

Yes

Reviewer Expertise:

genomics, bioinformatics

I confirm that I have read this submission and believe that I have an appropriate level of expertise to confirm that it is of an acceptable scientific standard.

# Reviewer report: Reviewer response for version 1

**DOI:** 10.21956/wellcomeopenres.25189.r105804

**Published:** 2024-11-07

**Authors:** Bernardo Santos

**Affiliations:** 1 Leibniz Institute for Evolution and Biodiversity Science, Berlin, Germany

In this contribution, the authors provide the full genome of an ichneumonid parasitoid wasp, *Ophion ventricosus*. The Ophioninae are a biologically interesting group in that most species are nocturnal, but a few are partially or entirely diurnal. The evolution of activity time and its relationship to host use and diversification is a topic of great potential interest to be studied in the group, and understanding the genes associated with this switch is made more feasible by the availability of high-quality genomes. In this context, the availability of yet another *Ophion* genome is a worthwhile contribution. The methods used to produce the genome are straightforward and well in line with previous hymenopteran genomes produced by the Darwin Tree of Life initiative. I can definitely provide a positive assessment of this study and I do not think it needs any further editing.

Are sufficient details of methods and materials provided to allow replication by others?

Yes

Is the rationale for creating the dataset(s) clearly described?

Yes

Are the datasets clearly presented in a useable and accessible format?

Yes

Are the protocols appropriate and is the work technically sound?

Yes

Reviewer Expertise:

Phylogenomics, systematics, evolutionary biology.

I confirm that I have read this submission and believe that I have an appropriate level of expertise to confirm that it is of an acceptable scientific standard.

# Reviewer report: Reviewer response for version 1

**DOI:** 10.21956/wellcomeopenres.25189.r100389

**Published:** 2024-10-14

**Authors:** Paulo Cseri Ricardo

**Affiliations:** 1 University of Sao Paulo, São Paulo, State of São Paulo, Brazil

The paper presents data on the genome assembly of the ichneumonid wasp *Ophion ventricosus*, a Palearctic parasitoid species distributed from Britain to the eastern limit of Iran. The main result is a final assembly comprising 436.80 Mb, organized into 621 scaffolds, with 16 chromosome-scale scaffolds accounting for 97.01% of the total sequence. A 27.71 Kb mitogenome was also assembled. These results were achieved using HiFi long reads combined with Hi-C mapping, the gold standard method to obtain chromosome-scale scaffolds. Assembly metrics indicate high quality (QV=52.0) and good completeness, with high k-mer completeness (99.97%) and suitable single BUSCOs value (92.6%). The protocols are well-described or referenced to ensure reproducibility. The manuscript is clear and concise, offering valuable information for studies on *Ophion* evolution and other hymenopteran parasitoids. I believe it is suitable for indexing in its current format.

Are sufficient details of methods and materials provided to allow replication by others?

Yes

Is the rationale for creating the dataset(s) clearly described?

Yes

Are the datasets clearly presented in a useable and accessible format?

Yes

Are the protocols appropriate and is the work technically sound?

Yes

Reviewer Expertise:

transcriptomics, genomics, and evolution of bee species

I confirm that I have read this submission and believe that I have an appropriate level of expertise to confirm that it is of an acceptable scientific standard.
